# Analysis of the Ink-Stream Break-Up Phenomenon in Continuous Inkjet Printing

**DOI:** 10.1021/acsomega.3c02790

**Published:** 2023-09-11

**Authors:** Takuya Otowa, Shigetaka Tsubouchi, Yuji Suwa

**Affiliations:** Research & Development Group, Hitachi, Ltd., 7-1-1, Omika, Hitachi, Ibaraki 319-1292, Japan

## Abstract

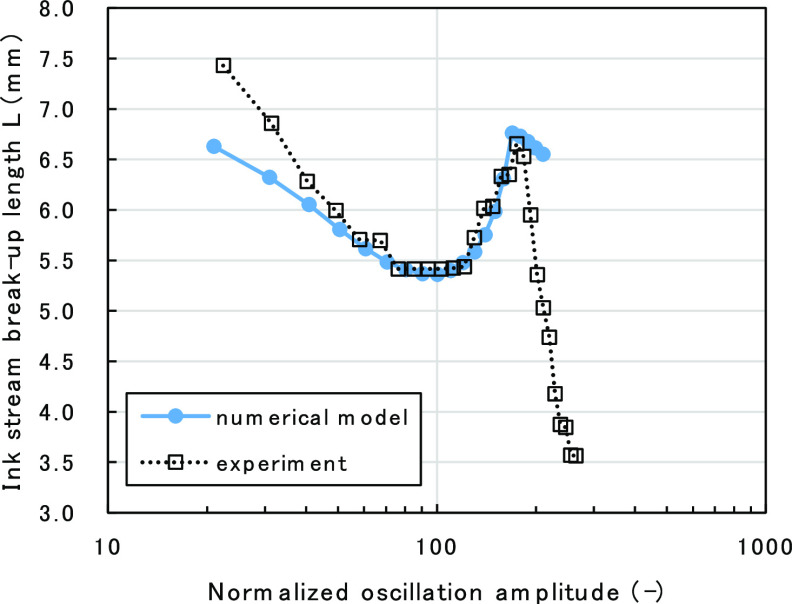

The ink-stream break-up phenomenon in continuous inkjet printers has been studied herein. A numerical model has been developed to reproduce and analyze the non-monotonic behavior of ink-stream break-up length (BUL) against the amplitude of piezo-actuator oscillation. That is, when the amplitude is increased, the BUL initially decreases to a local minimum point, then increases to a local maximum point, and finally decreases again. The developed model is split into two stages, first being the emergence of periodic “initial indentation” on ink stream caused by piezo-oscillation and the second being the growth of indentation. Finally, the calculated results of BUL against oscillation amplitude is compared with experimental data. We confirmed that the model well reproduces the characteristic of BUL and clarified the emergence mechanism of its local minimum and maximum points.

## 1 Introduction

Modern inkjet printers can basically be categorized into 2 types: drop-on-demand (DOD) printers and continuous inkjet (CIJ) printers according to their printing principle.^1^ As DOD printers can eject smaller ink droplets and print detailed images, they are widely used as desktop printers. Although the printed dot diameter is larger compared to DOD, a CIJ printer has the capability to print at a faster speed. Thus, the CIJ printer is widely used in industrial applications to mainly print traceability information onto packages of food, beverages, pharmaceuticals, etc.

**Figure 1:**
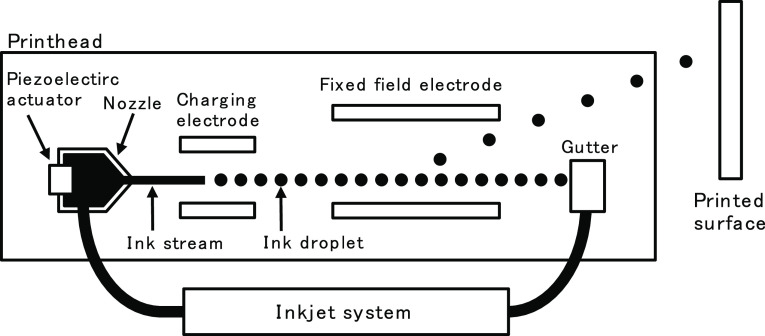
Schematic diagram of the CIJ printer.

The CIJ printer, shown in Figure 1, typically functions as follows. First, the ink is ejected from the nozzle, forming an ink stream. In order to produce homogeneous ink droplets at designated frequency, periodic oscillation (perturbation) is applied to the ink stream, typically using a piezoelectric actuator. At the point of ink-stream break-up where ink droplets are formed, the “charging electrode” is positioned and the electric field is selectively applied to each droplet, which in turn controls the charging amount of each droplet. The droplets then pass through a “fixed electric field”, which deflects the flying direction of droplets, according to the charging amount. Deflected droplets then land on the targeted surface to print alphanumeric characters. The un-charged droplets do not get deflected and are collected to be re-used in later printing.

**Figure 2:**
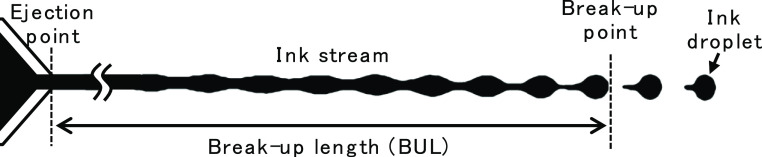
Schematic diagram of ink-stream break-up.

**Figure 3:**
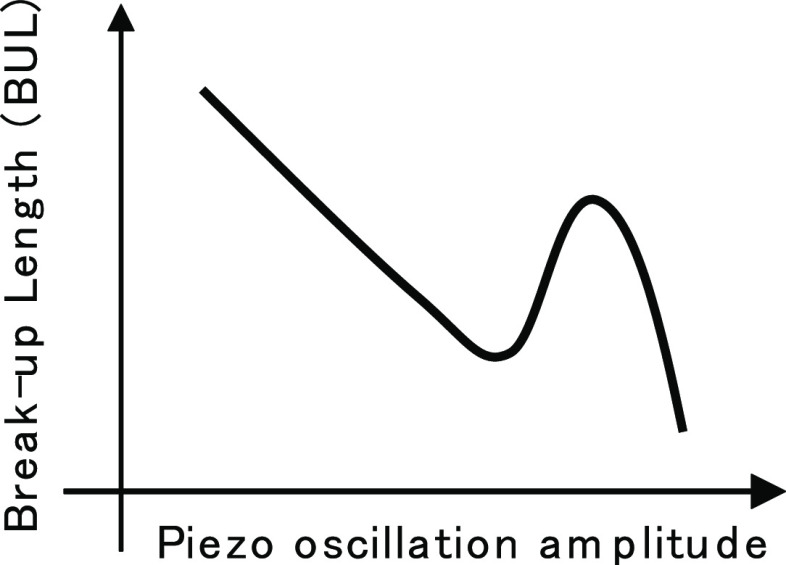
Typical non-monotonic behavior of BUL against piezo-oscillation amplitude.

Here, break-up length (BUL) is defined as the distance between the tip of the nozzle where ink is ejected and the ink-stream break-up point, as shown in Figure 2. It is known that the BUL is dependent on the amplitude of the piezo-perturbation and exhibits non-monotonic behavior.^2,3^ That is, when the amplitude is increased, the BUL initially decreases to a local minimum point, then increases to a maximum point, and finally decreases again, as shown in Figure 3. BUL behavior may also vary depending on other factors such as temperature, ink-stream velocity, ink density, ink viscoelasticity, ink chemical composition, etc. If the break-up point of ink stream exceeds the charging electrode range due to BUL fluctuation, ink droplets will not get charged properly, resulting in print failure. When designing the CIJ printer and ink composition, understanding the BUL phenomenon and its dominant factors is the key to control BUL behavior and thus achieve robust printing.

When designing the CIJ printer and ink composition, understanding the BUL phenomenon and its dominant factors is the key to control BUL behavior and thus achieve robust printing.

Under these circumstances, BUL behavior has been the target of research for many years from both experimental^4−6^ and computational^7,8^ stand points. However, the focus of the research of BUL behavior against amplitude has mainly been the range up to the BUL local minimum point.^9,10^ To the authors’ knowledge, there has not been a model that explains the emergence of both local minimum and maximum points of BUL. Hence, there is still a lack of understanding of the phenomenon as a whole, despite the widespread practical application of CIJ.

In this paper, we present a simple numerical model that well reproduces the local minimum and maximum points of BUL and explains its emergence mechanism. We first report the results of experimental measurements of ink-stream BUL at the respective oscillation amplitude. Next, we present the numerical model to explain the origin of non-monotonic behavior of the CIJ ink-stream break-up phenomenon and thus the BUL fluctuations. Finally, the calculated results of BUL fluctuations were compared with the experimental results to verify the validity of the numerical model.

## 2 Experimental Measurement of BUL

Ink-stream BUL fluctuations have been measured experimentally using the CIJ printer. Figure 1 shows the diagram of the printhead used in this study. Using a nozzle with a radius of *a* = 33 μm, the oscillation wavelength was adjusted to λ = 290 μm, as according to Rayleigh^11^ λ ∼ 9*a* is the preferred condition with the most rapid growth of ink droplets. In accordance with the piezo-oscillation frequency used, the average velocity was set to *v*_0_ = 20 m/s.

The sample ink, consisting of a metal complex dye and vinyl resin in 2-butanone solvent, was prepared. The ink viscosity was adjusted to 3.0 mPa s at 20 °C, which was measured using a Brookfield LVDV-II-PCP viscometer. The applied voltage on the piezo-electric actuator was adjusted to measure the oscillation amplitude dependency of the ink-stream length. In order to capture the still image of ink stream with a conventional camera to extract ink-stream BUL, a strobe light source with the blinking frequency equivalent to the piezo-oscillation was used as a backlight. The ink-stream image was captured at room temperature.

**Figure 4:**
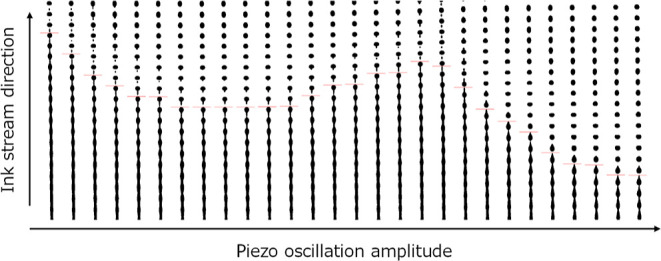
Captured ink-stream images against piezo-oscillation amplitude, with break-up point indicated in red.

**Figure 5:**
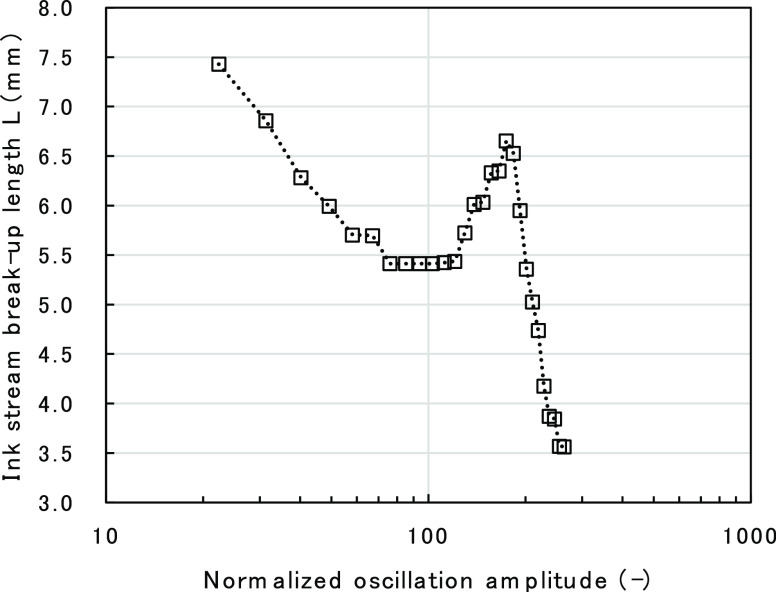
Oscillation amplitude dependency of ink-stream BUL.

Figure 4 shows the transition of captured ink-stream images against the piezo-oscillation amplitude, with the break-up point indicated in red. Ink-stream BUL was extracted from the images and plotted against the amplitude, as shown in Figure 5. Local minimum and maximum points of BUL were successfully observed in the measured amplitude range. The amplitude axis was normalized at the local minimum point of BUL to compare the results against the calculated numerical model in Section 4.

## 3 Numerical Calculation of BUL

### 3.1 Qualitative Explanation of the BUL Numerical Model

Our newly constructed BUL numerical model is focused on reproducing the non-monotonic behavior of ink-stream BUL against the piezo-oscillation amplitude. This is enabled by calculating the time from the point when the ink stream is ejected from the nozzle with piezo-oscillation to the point when the ink breaks up into individual droplets in the simplest way possible while retaining the essence of the phenomenon. After much consideration, we have come to the conclusion that it is best to describe the process in two stages as the dominant factor transitions. The first stage is the emergence of “initial indentation” on the surface of ink stream. The second is the growth of the indentation depth, which finally leads to ink-stream break-up. The first stage is dominated by the relative mass advection within ink stream caused by the piezo-oscillation and the second stage is dominated by the surface tension of ink stream.

**Figure 6:**
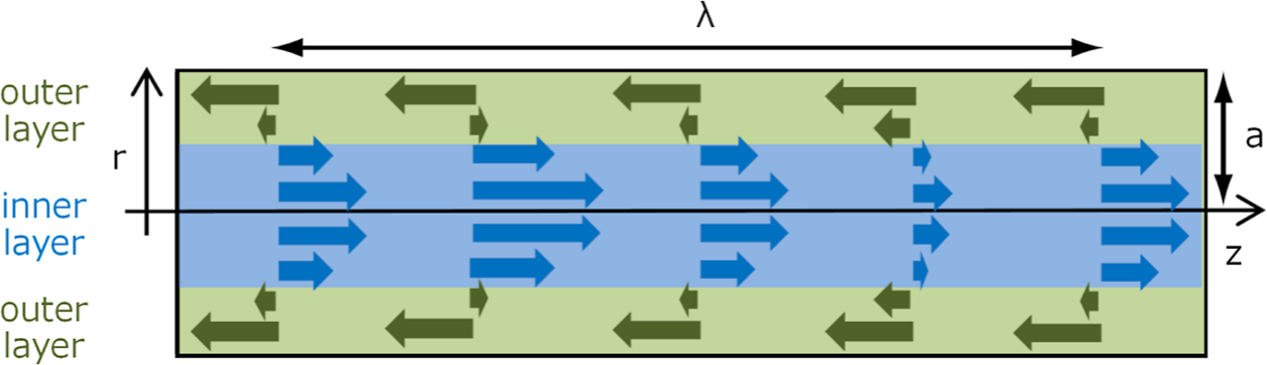
Schematic picture of the relative velocity distribution inside the ink stream at *t* = 0.

The first stage, the emergence of “initial indentation”, can be explained by considering the superposition of two different velocity distributions within the ink stream just after the ink is ejected from the nozzle. One is the velocity distribution along the radius of the ink stream, caused by nozzle wall friction, known as the Hagen–Poiseuille flow. Two is the velocity distribution along the traveling direction of the ink stream, originated by the piezo-oscillation. Figure 6 shows the velocity distribution of the ink stream. The figure is described in relative velocity within the ink stream. Therefore, the “outer layer” of ink stream, which is slower than the average velocity, is represented as opposite (right to left) to the traveling direction of the ink stream (left to right). Immediately after ink ejection, the ink is in the shape of a straight cylinder. As the ink stream relatively moves over time based on the velocity distribution within the ink stream, certain parts of the ink stream become thicker and others become thinner thus creating thickness distribution along the traveling direction. Although the relative velocity distribution quickly relaxes to zero due to the viscous behavior of the ink fluid, the thickness distribution of ink stream remains. Here, the thinnest part of the ink stream at the end of the first stage is called the “initial indentation”. As the initial indentation is the result of the piezo-oscillation, the indentation is formed periodically on the ink stream. The interval of the indentation is equivalent to the piezo-oscillation wavelength λ.

The second stage is the growth process of the indentations using equation derived by Rayleigh^11^ and Weber.^12^ We shall note that their theory explains the growth of indentation caused by infinitesimal fluctuations, without piezo-oscillation. However, the only difference here is that we start from the indentation with finite size, and we therefore assume that their theory applies to our system as well. When the indentation depth reaches the nozzle radius *a*, ink-stream break-up occurs, forming ink droplet as a result.

### 3.2 Construction of the BUL Numerical Model

In this section, we explain the model by showing the numerical equation in detail. Here, we state the time range for the first stage to be 0 ≤ *t* ≤ *t*_0_ and the second to be *t*_0_<*t* ≤ *t*_b_, while *t* = 0 is the point when the ink is ejected from the nozzle and *t* = *t*_b_ is the point when ink-stream break-up occurs. *t* = *t*_0_ is the time when transitioning from the first stage to the next, and its value will be discussed in the later part of this section.

By having the coordinate of the direction of ink-stream flow on the *z*-axis and the coordinate of the ink-stream radius on the *r*-axis, the initial relative velocity distribution *v* at *t* = 0 in the first stage can be expressed as
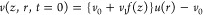
1
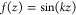
2
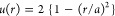
3where *v*_0_ is the average flow velocity of the ink stream, *v*_1_ is the amplitude of the flow velocity added by the piezo-oscillation, *a* is the nozzle radius, and *k* is the piezo-oscillation wavenumber, equal to 2π/λ. *f*(*z*) is the piezo-oscillation waveform, assumed to be the sine wave. *u*(*r*) is the velocity profile in the radial direction, approximated by the Hagen–Poiseuille flow. Space range calculated in this model is 0 ≤ *z* ≤ λ for the *z*-axis and 0 ≤ *r* ≤ *R* for the *r*-axis. *R*(*z*,*t*) is the ink-stream radius distribution that can be expressed as follows at *t* = 0
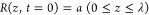
4

As the indentation is formed periodically due to the piezo-oscillation with the interval of λ, we can set a periodic boundary condition for the *z*-axis that can be described as follows
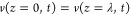
5
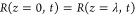
6

Figure 6 is a schematic representation of the relative velocity distribution *v*(*z*,*r*,*t*) at *t* = 0 in the ink stream defined by eq 1. The direction of the ink stream in the figure is left to right, along the *z*-axis.

Here, an approximation is introduced to calculate how the ink-velocity distribution slows down over time.
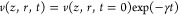
7Here, γ is the relative velocity decrement parameter. Although the velocity decrement is a complex phenomenon caused by both ink viscosity and surface tension, they are approximated in this model by the single parameter γ. The value of γ will be determined as an ink parameter, based on comparison with the experimental results.

There are several ways for determining *t*_0_, the time transitioning from the first stage to the next. One way is to set fixed *t*_0_ as the time when exp (−γ*t*) in eq 7 becomes sufficiently small. Another possible method is to set *t*_0_ as the time when the chronological change in the initial indentation depth becomes sufficiently slow. Considering the fact that the time required for the first stage to complete is sufficiently shorter than that of the second stage, calculating the precise time of transition will have a small effect on the calculation result of the ink-stream length as a whole. Therefore, we judged that it is reasonable to set the fixed time for *t*_0_.

**Figure 7:**
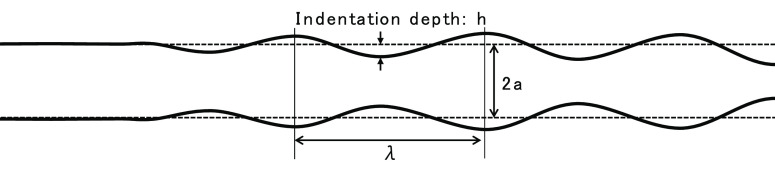
Schematic diagram of the indentation depth of ink stream.

By taking the relative velocity distribution and its relaxation process into account, we can derive ink-stream thickness *R* distribution at *t* = *t*_0_ by calculating the mass balance equation of ink stream, as described in eq 8.
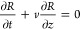
8In the *R* distribution at *t* = *t*_0_, the minimum point is defined as the “initial indentation”, and we define its depth as *h*_0_. Here, the indentation depth *h* in general is defined as the difference between the nozzle radius *a* and the radius at the indentation point of ink stream, as described in Figure 7.

The second stage is the growth of the indentation depth *h*. Rayleigh^11^ showed that for the ink stream of initial indentation depth *h*_0_ and amplitude growth rate μ, the indentation depth *h* at *t* in our system is given by
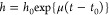
9

According to Rayleigh,^11^ the amplitude growth rate μ is dependent on the surface tension Γ and the density ρ of the fluid. Weber^12^ points out that μ is also dependent on the fluid viscosity η. The theoretical equation is provided by Weber as
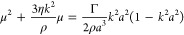
10Here, μ will be the ink parameter determined according to the experimental results and will be compared with the theoretical value in the next section. Ink-stream break-up occurs when the indentation depth *h* reaches the nozzle radius *a*. From eq 8, the ink-stream break-up time *t*_b_ is given by
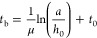
11

For the ink stream of average velocity *v*_0_, BUL *L* can be derived by
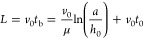
12In summary, ink parameters γ and μ will be determined according to experimental results comparison, and the fixed value of *t*_0_ will be determined in accordance to γ. *v*_0_, *a*, and λ will be given from the experimental condition. Piezo-oscillation amplitude dependency will be verified by changing the parameter *v*_1_.

## 4 Results and Discussion

**Figure 8:**
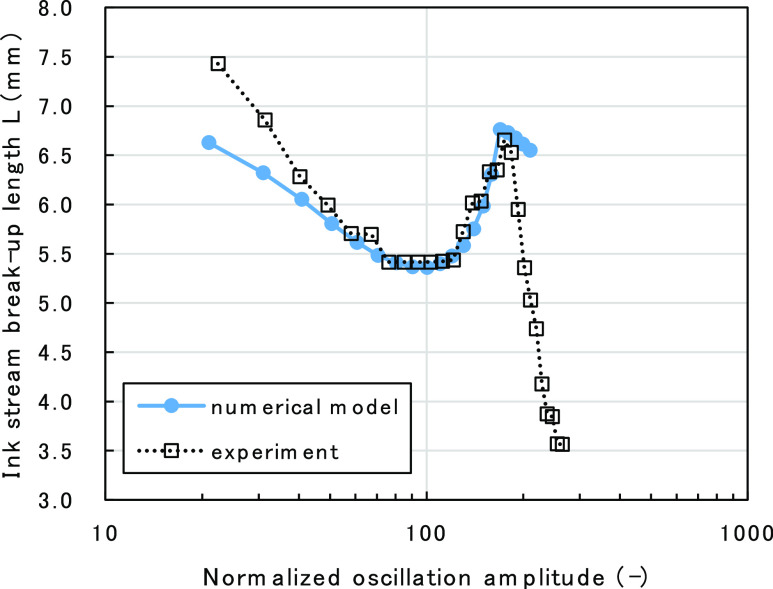
. Comparison of ink-stream BULs between the numerical model and experimental data.

In this section, we present the calculated results of the numerical model constructed in Section 3.2 and verify the validity of the numerical model by comparing it with the experimental results. Figure 8 shows the calculated results of oscillation amplitude dependency of ink-stream BUL, compared with actual experimental data. Oscillation amplitude on the *x* axis was normalized with the amplitude at the local minimum point of ink-stream length to compare the data with experimental results.

In the calculated model, the amplitude growth rate, which determines the absolute value of BUL *L*, was adjusted to μ = 6.4 × 10^3^ s^–1^. The theoretical value of μ, dependent on the physical properties of ink (viscosity η = 3.0 mPa s, surface tension Γ = 2.5 × 10^–2^ N/m, and density ρ = 900 kg/m^3^) and nozzle radius *a* = 33 μm was calculated by eq 10 to be μ = 8.0 × 10^3^ s^–1^. The value of μ in the numerical model is well in the reasonable range. The relative velocity decrement parameter γ, also dependent on ink properties, was set to γ = 0.14 μs^–1^. Transitioning time from the first stage to the next was set to *t*_0_ = 15 μs, as exp(−γ*t*_0_) ∼ 1/*e*^2^ and growth of initial indentation depth at *t* > *t*_0_ are well negligible.

The calculated results well reproduced the basic characteristics of the measured ink-stream BUL when the piezo-amplitude is increased. That is, BUL initially decreases to a local minimum point, then increases to a maximum point, and finally decreases again.

In the experimental data, the ink-stream BUL decreases rapidly (a) before the local minimum point and (b) beyond the maximum point, when compared with the calculated value. In order to improve the quantitative analysis, it may be necessary to improve the approximation of the motion of the fluid until the initial relative velocity distribution of the fluid relaxes in the first stage. In addition, strictly speaking, the actual velocity profile *u*(*r*) deviates from eq 3 because the CIJ nozzle have a short nozzle length compared to the nozzle radius, which causes the boundary layer to be unable to fully develop even at the tip of the nozzle.^10^ It may be necessary to implement the precise velocity profile to improve the model precision.

**Figure 9:**
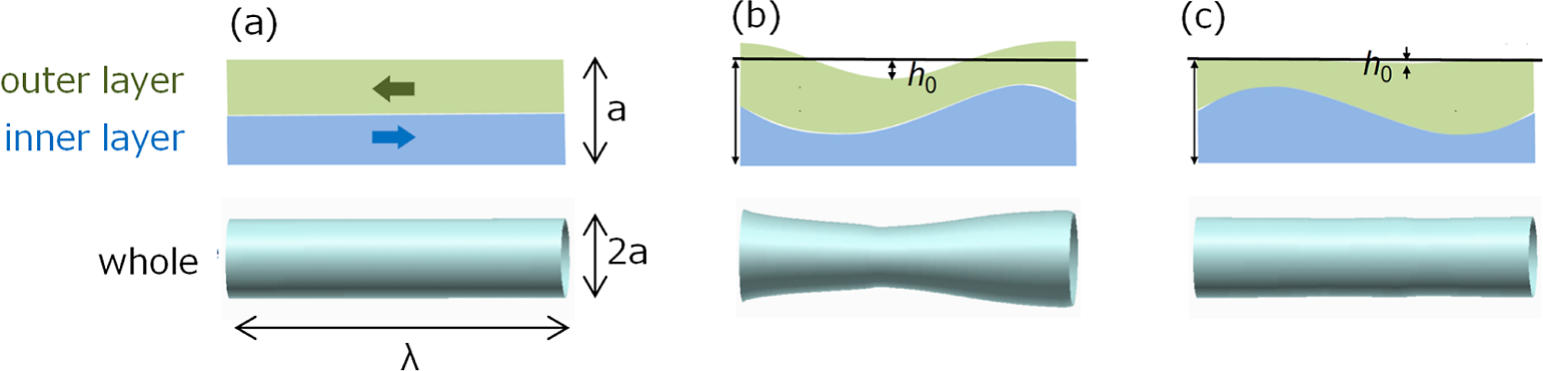
Schematic diagram of ink-stream thickness distribution (a) at *t* = 0, (b) at *t* = *t*_0_ with amplitude near the BUL local minimum point, and (c) at *t* = *t*_0_ with amplitude near the BUL maximum point.

Despite the simplification of the model mentioned above, the numerical calculation performed here has reproduced the existence of the local maximum point and clarified its emerging mechanism. Figure 9 shows schematically how the oscillation amplitude affects the stream thickness distribution. Figure 9a shows the stream thickness distribution at *t* = 0 just after ink ejection. Although there is a non-uniform velocity distribution, its cylindrical shape has not been affected yet. Figure 9b shows the thickness distribution at *t* = *t*_0_ when the oscillation amplitude condition is near the local minimum point of ink-stream BUL. The outer layer and the inner layer within the ink stream gather in different parts of the stream, creating a large initial indentation as a whole. The deeper the initial indentation depth *h*_0_ is, the shorter the BUL will become, as it takes shorter time for the indentation depth to reach *h* = *a* in the second stage, as expressed in eq 12.

As the oscillation amplitude is increased, the initial relative velocity difference will become greater. As each ink layer travels farther, there will be a range in amplitude in which the ink movement of the outer layer and the inner layer would complement each other, ultimately forming very shallow initial indentation depth as a whole, as described in Figure 9c. As a result, BUL becomes longer compared to the condition illustrated in Figure 9b. A further increase in the oscillation amplitude will cause the initial indentation to become larger, thus creating a maximum point.

## 5 Conclusions

In this paper, a numerical model has been developed to reproduce and analyze the non-monotonic behavior of ink-stream BUL against the amplitude of piezo-oscillation. In order to reflect the transition of the dominant factor during the process, the developed model was split into two stages: the first being the emergence of periodic “initial indentation” on ink stream caused by piezo-oscillation and the second being the growth of the emerged indentation. BUL was derived by multiplying the average velocity speed of ink stream and the time required for the ejected ink stream to break up, which was calculated by the model.

Finally, the calculated results of BUL against the oscillation amplitude were compared with experimental data. We confirmed that the model well reproduces the non-monotonic characteristic of BUL and clarified the emergence mechanism of its local minimum and maximum point. The numerical model developed in this paper is very important for understanding the BUL fluctuation behavior to achieve a stable CIJ printer operation. The correlation between ink parameters γ and μ used in the numerical model and the physical properties of ink such as viscosity η and surface tension Γ will be further studied in the future.
